# Public perception vs ecological quality status: Examining the ecological restoration of the Congost River's Environment

**DOI:** 10.1016/j.heliyon.2024.e34615

**Published:** 2024-07-14

**Authors:** Quim Zaldo-Aubanell, Antoni Mas-Ponce, Guiu Asbert, Berta Clota, Manel Isnard, Lorenzo Proia, Albert Bach, Sònia Sànchez Mateo

**Affiliations:** a BETA Technological Center, University of Vic - Central University of Catalonia (UVic-UCC), Vic, Spain; b Forest Science and Technology Centre of Catalonia (CTFC), Solsona, Spain; c Geography Department, Autonomous University of Barcelona (UAB), B building, UAB Campus, 08193, Bellaterra (Cerdanyola del Vallès), Barcelona, Spain; d Consorci Besòs Tordera, Avinguda Sant Julià, 241, 08403, Granollers, Catalonia, Spain; e Centre de Recerca Ecològica i Aplicacions Forestals, Cerdanyola del Vallès, Catalonia, Spain; f Autonomous University of Barcelona (UAB), Bellaterra (Cerdanyola del Vallès), 08193, Barcelona, Spain; g Fundació RIVUS, Avinguda Sant Julià, 241, 08403, Granollers, Catalonia, Spain; h Institute of Environmental Science and Technology (ICTA), Autonomous University of Barcelona (UAB), Z building, ICTA-ICP, Carrer de les columnes, UAB Campus, 08193, Bellaterra, Barcelona, Spain

**Keywords:** Ecological indicators, Public perception, Sociodemographic groups, Survey analysis, Environmental restoration strategies, Environmental attitudes

## Abstract

This study examines the public's perceptions of the ecological restoration of the Congost River over the past thirty years, focusing on the period between 2010 and 2022. We conducted a survey of 112 river users across five key zones identified through a pilot study for their high pedestrian density, aiming to analyse how different sociodemographic groups perceive the river's ecological state. A structured questionnaire was distributed along both sides of the river to engage a diverse range of individuals typically utilizing the river environment. The collected data were analysed using regression models and Mann-Whitney U tests to assess differences between groups, with Bonferroni adjustments applied to control for multiple comparisons.

The results reveal a broad increase in appreciation for the river since 2010, alongside measurable ecological improvements supported by scientific data. Despite these positive changes, a majority of surveyed users remain sceptical about the river's recovery, with less pronounced scepticism among older respondents, those with higher education, and environmental volunteers. These groups' perceptions align more closely with empirical evidence, highlighting the influence of sociodemographic factors on environmental awareness.

Individuals living closer to natural settings and frequent river visitors were found to be more attuned to changes in the river's environment, particularly in aesthetic and sensory aspects. The study underscores the persistence of a perceptual gap between scientific assessments of ecological health and public sentiment, emphasizing the complex relationship between community perceptions and objective environmental indicators. These insights underline the complex relationship between community perceptions and objective environmental indicators, reflecting a broader trend in environmental awareness and the importance of factual communication in ecological issues.

## Highlights

• Explores 30 years of Congost River ecology vs. perceptions from 2010 to 2022.
• Unravels shifts in user opinion across varied sociodemographic sectors.
• Users value the river, but many do not recognize ecological advancements.
• Age, education, and volunteering influence alignment with scientific findings.
• Disconnect between managers and users due to lack of communication or mismatched metrics.

## 1 Introduction

Rivers, frequently termed the “arteries of our planet”, play a paramount role in supporting life and moulding civilizations. Their relevance extends beyond mere water provision to acting as sanctuaries for biodiversity, regulating our climate, and serving as reservoirs of cultural heritage [[1], [2], [3]]. Yet, the resonance of riverine ecosystems is juxtaposed with alarming global trends: freshwater habitats, bearing the brunt of anthropogenic pressures, have witnessed an average 84 % decline in species populations within less than 50 years, as reported in the most recent Living Planet Report [4]. This rapid decline not only endangers ecosystems but also underscores the acute need for informed and inclusive river management strategies [5].

Public perceptions of these natural spaces often diverge from their actual ecological status. Such perceptions are shaped by a complex interplay of personal preferences [6], depth of knowledge [7], and immediate sensory experiences [[8], [9], [10]]. Rivers, given their historical, ecological, and cultural significance, present a unique challenge in this matrix [11]. While scientific evaluations offer an empirical perspective, truly sustainable river management seeks to align these objective insights with the sentiments and perceptions of local communities.

The urgency to integrate public opinions into environmental policies has gained momentum globally [[12], [13], [14], [15]]. This integration not only bolsters policy frameworks [16] but also ensures their relevance and longevity by aligning them with grassroots aspirations [17]. However, in the realm of rivers, a significant gap persists between perceived and quantified environmental quality, suggesting a disconnect that may undermine the effectiveness of restoration policies [10,[18], [19], [20], [21]].

To address this disconnect, our study emphasizes the importance of integrating public perceptions with long-term biological monitoring efforts. We aim to bridge the existing gap between scientific assessments of river health and the community's subjective perceptions. This integration seeks to enhance the applicability and acceptance of environmental policies by ensuring they resonate more closely with community experiences and expectations. Our research delves into a three-decade-long study of the Congost River, highlighting the pronounced ecological advancements documented by scientists and contrasting them with the local community's perceptions. This approach seeks to determine whether improvements in river health, as evidenced by long-term ecological data, resonate with the community's experience and awareness.

Our exploration is guided by three central research questions:

- How have perceptions regarding the Congost River's environment evolved from 2010 to 2022?
- How do perceptions regarding the Congost River's environment vary among different sociodemographic segments?
- Is there an alignment between the scientifically monitored ecological quality status and the prevailing public perceptions of the river?

To shed light on these questions, we harness insights from the comprehensive 2022 Survey, a tool that not only probes diverse environmental sentiments but also facilitates a historical comparison with its 2010 predecessor.

## 2 Methodology

### 2.1 Study area: the Congost River's environment

The Besòs River Basin, spanning a drainage area of 1038 km^2^, lies within the cityscape of Barcelona, Spain. Nested within this basin, the Congost River Basin covers 225 km^2^ and holds unique environmental and socio-cultural significance. Merging with the Mogent River at Montmeló, it contributes to the formation of the Besòs River. Our study narrows its lens on a 6.7 km stretch of the Congost River coursing through Granollers, designated as the “Congost River's environment”.

The river exhibits Mediterranean hydrological patterns, with marked seasonal variations. Historically influenced by rapid urbanization and industrial pressures, the river underwent stark ecological degradation. But post-1974, a series of initiatives encompassing river restoration, sanitation, urban planning, and environmental education paved the way for its gradual recovery [22].

#### 2.1.1 Can Cabanyes natural area

Can Cabanyes, located in the southern part of Granollers near the Congost River and adjacent to the Circuit de Catalunya (a major motorsport race track), has experienced significant ecological rehabilitation. This natural area, once degraded and polluted, is now a revitalized green space. Key to its transformation was the development of adjacent industrial areas, which led to the acquisition of about 8 ha for environmental restoration.

This regeneration involved planting two thousand oak trees, establishing a rest area, and constructing a small environmental education centre. A one-hectare wetland was also created for effluent treatment, alongside leisure paths near the river. These efforts align with the broader environmental recovery within the Congost River's environment, illustrating the positive impact of integrated urban and environmental planning in reversing ecological degradation [22].

### 2.2 Ethical approval

This study was approved by the Fundació RIVUS, Spain, with ethics approval reference OBT–2022-001 on September 10, 2022. Informed consent was obtained from all the participants. Our study strictly adheres to the EU General Data Protection Regulation (GDPR) to ensure the highest standards of privacy and data protection for participants. Although our research does not involve clinical trials, we align our practices with the ethical principles outlined in the Declaration of Helsinki, emphasizing respect for all participants, their autonomy, and the confidentiality of their responses. The questionnaires were anonymized, and patients were free to opt out of participation in the study whenever they were uncomfortable.

### 2.3 Evolution of the ecological status of the Congost River. Monitoring results from the period 1995–2021

To gauge the ecological progression of the Congost River over nearly 30-year span, we harnessed data from two distinct sources.

Firstly, we explored the long-standing biological indicators, with a particular emphasis on the Iberian Bio-Monitoring Working Party (IBMWP) index. Grounded in macroinvertebrates communities, the IBMWP index is a renowned and robust measure of water quality [23]. Owing to its comprehensive coverage over the years (1995–2021), this index emerged as a prime candidate to elucidate the biological trajectory of the river.

In tandem, we also assimilated findings from pertinent local and regional studies conducted on-site. This facilitated the incorporation of both semi-quantitative metrics and qualitative insights, particularly bioindicator species. Such multifaceted presented a holistic picture of the river's ecological transformation [[24], [25], [26], [27]].

### 2.4 Evaluation of public perceptions: 2022 surveys and data gathering

**Fig. 1:**
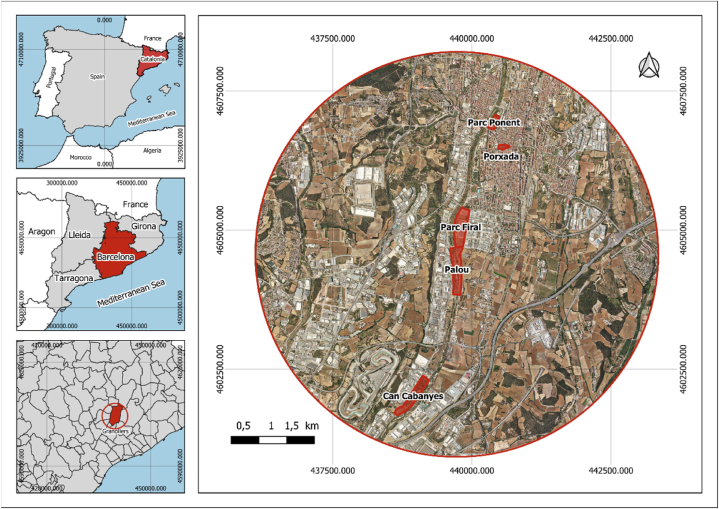
Sampling zones within the municipality of Granollers (Barcelona, Spain). All the zones, with the exception of la Porxada, are located in proximity to the Congost River.

In our investigation of public perceptions, we utilized data from the 2022 Survey, which was divided into three distinct segments: a record of participants' sociodemographic information, a replication of the 2010 Survey, and a series of thirty Likert-scaled statements evaluating environmental attitudes (refer to Fig. S1 in the Supplementary Materials). The sampling process was executed during varied time intervals and spanned a range of geographical locations (see Fig. 1), as guided by a preliminary pilot study (n = 30). We received feedback from 112 individuals associated with the Congost River's environment. For queries presenting ambiguous choices such as “NA” or “I do not want to answer”, significance calculations were performed excluding these particular responses.

Recruitment of interviewees was meticulously conducted along both sides of the river, at locations where pedestrian and cycling paths intersect. Based on a pilot study, these specific zones were identified as hotspots of high pedestrian density and strategically selected to maximize the likelihood of engaging a diverse sample of river users. To ensure a representative and unbiased sample, individuals using electric scooters and bicycles were explicitly excluded from the survey due to their transient nature and potential lack of sustained engagement with the river environment.

Most critically, our approach to data collection emphasized immediate and consensual participation. Interviewers approached pedestrians directly, explaining the purpose of the study and asking for their willingness to participate. Upon receiving verbal consent, surveys were administered and completed on the spot, ensuring that each participant fully understood and agreed to engage in the study at that moment. This direct engagement method not only facilitated real-time data collection but also upheld the highest standards of ethical research practices by respecting participants’ autonomy and willingness to contribute. Additionally, careful measures were taken to avoid surveying any individual more than once, preserving the integrity of the data collection process and ensuring that each response was uniquely accounted for.

In our exploration of shifts in public perception, the 2022 Survey incorporated identical queries as those presented in the 2010 survey. Nevertheless, one query was excluded due to the 2010 Survey responses being presented in an aggregated manner, complicating statistical analysis. Consequently, eight out of the nine questions from the 2010 Survey were replicated (refer to Fig. S1b in the Supplementary Materials).

The third segment of the 2022 Survey employed a five-point Likert scale to gauge environmental sentiments (see Fig. S1c). Participants were prompted to indicate their level of concurrence, ranging from “strongly agree” to “strongly disagree”. Surveys utilizing the Likert scale have found extensive application in social research [28]. Similarly, psychological assessments frequently incorporate a Likert response scale, facilitating structured response options and aiding in the evaluation process [29]. Drawing from previous studies [16,27], the statements were categorized into sociocultural, well-being, biodiversity, environmental impact, and political implications sectors. An exhaustive analysis, encompassing both concrete and abstract perceptions, was conducted.

The amalgamation of thirty statements and the five distinct agreement levels offered an exhaustive range of potential statement types. Simultaneously, this approach addressed possible biases inherent to Likert scale surveys, such as employing a five-point response for reasons not rooted in psychometrics, like clarity or simplicity [29,30].

### 2.5 Sociodemographic profile of the sample (n = 112)

To accentuate contrasts in perceptions, we systematically documented sociodemographic nuances including age, gender, education, employment status, household income, and other pivotal variables (see Fig. S1a). Each variable was selected for its relevance to our central research question, which seeks to explore perceptual differences among various sociodemographic segments of river users. This selection was informed by their recurrent use in environmental perception studies, ensuring our approach aligns with established methods for examining demographic influences on environmental attitudes [31,32].

An overview of our sample is detailed in Table 1, reflecting the diversity and representativeness of the participants involved in the study.

**Table 1:** Summary of sociodemographic profile of the sample. “M”, stands for mean, while “SD” stands for standard deviation.

Sociodemographic variable	Value	Sociodemographic variable	Value
**Age**:	M: 51; SD: 17.76	**Employment status**:	
**Gender**:		not seeking employment	4.46 %
female	61.60 %	seeking employment	8.03 %
male	37.50 %	retired	33.03 %
non-binary	0.89 %	part-time working	13.39 %
**Education level**:		full-time working	41.07 %
high school	25.00 %	**Household income:**	
technical/vocational	25.0 %	0–21.000 €	25.00 %
college degree	3.57 %	21.001–40.000 €	22.32 %
graduate degree	29.46 %	>40.000 €	15.17 %
master's or higher	8.03 %	Prefer not to say	37.50 %
no education	6.25 %	**Municipality:** Granollers	86.60 %
other	2.67 %	**Access to river's environment**	
**Children** < **18 years**:		on foot	1.78 %
yes	80.35 %	bike, scooter or similar	89.28 %
no	19.26 %	car or other motor vehicle	8.92 %
**Residence surroundings**:		**Num. of weekly visits**:	M: 3.59; SD:2.67
<30 % natural environment	47.32 %	**Num. years familiar with:**	M: 13.04; SD:14.17
30–50 %	35.71 %	**Environmental volunteering participation:**	
> de 50 %	16.96 %	no	94.64 %
		yes	2.67 %
		yes, but not currently	2.67 %

### 2.6 Statistical analysis

Statistical analyses were conducted using the R programming environment (version 4.0.2). The initial step involved a thorough examination of the data using standard descriptive statistics, which included measures of central tendency summaries and frequency tables. For quantitative and ordinal variables, linear regression modelling was employed. Prior to modelling, we confirmed that the variables met essential assumptions: linearity, independence (as verified by the Durbin-Watson test), homoscedasticity (as determined by the Breusch-Pagan test), and normality of residuals (evaluated using the Shapiro-Wilk test).

For dichotomous and nominal sociodemographic variables, we opted for the Kruskal-Wallis test as a non-parametric alternative to ANOVA. The decision to use Kruskal-Wallis test was driven by the violation of the normality assumption, as evidenced by the results from the Shapiro-Wilk test on several of our variables. Nonetheless, we confirmed variance homogeneity (via the Levene's test) and verified shape consistency across groups, ensuring the test's suitability. Research indicates that for five-point Likert items, parametric and non-parametric procedures yield similar results, highlighting their comparable power [33].

In cases where significant differences were identified via the Kruskal-Wallis test, post-hoc pairwise comparisons were carried out utilizing the Mann-Whitney *U* test. This method proves especially advantageous with ordinal data or absent naturally defined measurement units, like attitude or perception scales [34]. Importantly, the U statistic derived from the Mann-Whitney test as produced by our implementation in R apply to the first group we input (referred to as Group 1 in our analyses). Therefore, the interpretation of the Mann-Whitney statistic offers insight into the likelihood of a randomly selected observation from Group 1 scoring higher than one from Group 2 [35].

To quantify these differences, we leaned on the Mann-Whitney U statistic to determine the effect size. By rescaling the U statistic, dividing it by its maximum value (product of the sample sizes of the two groups), we derived the “common language effect size” (CLES) or the “probability of superiority” [[36], [37], [38]]. The interpretation of CLES is conditional: if CLES >0.5, the result directly conveys the probability that a randomly chosen observation from Group 1 will score higher than another from Group 2. Conversely, if CLES <0.5, the likelihood is that an observation from Group 1 will score lower than one from Group 2.

Given the potential pitfalls of multiple comparisons, and after testing for less conservative procedures including Holm and Benjamini & Hochberg [39,40], we applied the Bonferroni correction to control for the increased risk of Type I errors [41]. However, dealing with multiple independent variables, each with multiple levels, we risked adjusting a very stringent significance level, potentially leading to a high Type II error rate.

Acknowledging critiques against the excessive conservatism of Bonferroni correction in extensive simultaneous testing scenarios [42], we implemented strategic measures. Similar to other research, we partitioned tests into separate groups [43], in our case, considering each independent variable as separate families of comparisons with their own set of tests.

In our findings, we present both unadjusted and Bonferroni-adjusted p-values. This dual approach serves a twofold purpose. Firstly, by reporting unadjusted p-values, we align with the perspective that some research contexts, especially exploratory ones, benefit from a less stringent approach, allowing for the generation of hypotheses for further investigations [44]. Secondly, given the multiple comparisons within our hypothesis-driven study, we also recognize the importance of the Bonferroni correction in rigorously managing the risk of Type I errors [45]. This refined approach strikes a balance between false positive control and test integrity [42]. Ultimately, our choice to report both types of p-values underscores our commitment to methodological transparency and robustness amidst the ongoing debates in the scientific community [44].

Comparisons of nominal variables were conducted using chi-square tests of independence. All tests satisfied the assumption of having sufficiently large expected cell frequencies. For two by two contingency tables, Yate's continuity correction was applied to improve the robustness of the chi-square approximation [46]. All tests were performed with a significance threshold set at *p value* < 0.05.

## 3 Results

### 3.1 Evolution of the ecological status of the Congost River. Monitoring results from the period 1995–2021

**Fig. 2:**
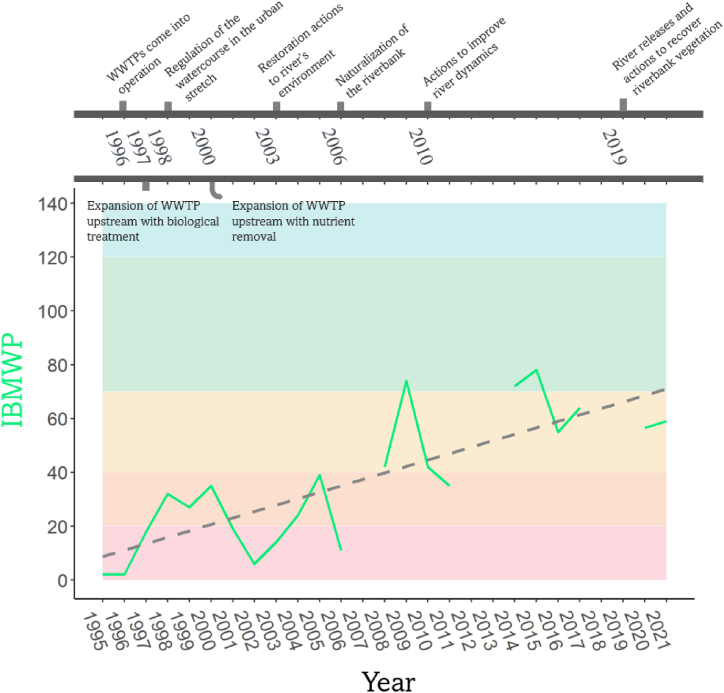
Ecological Status of the Congost River (1995–2021) using IBMWP Index. The dotted line indicates the linear regression analysis from 1995 to 2021 based on the IBMWP values: y=0.0065x; p-value = $6.49\times 10^{-6}$; R2 = 0.63. The timeline highlights environmental interventions in the Congost River's environment. The background color-coding represents ecological quality categories: poor (red, 0–20), bad (orange, 21–40), mediocre (yellow, 41–70), good (green, 71–120), and very good (>120). IBMWP data for certain years is unavailable. (For interpretation of the references to color in this figure legend, the reader is referred to the Web version of this article.)

Fig. 2 presents a linear regression model detailing the evolution of the IBMWP index over the period from 1995 to 2021 (y=0.0065x; *p-value* = $6.49\times 10^{-6}$; R^2^ = 0.63). The figure includes a timeline at the top, which chronologically lists the environmental interventions undertaken along the Congost River's and its environment. The IBMWP index is visually represented using color-coded intervals to indicate ecological quality categories: poor (red, 0–20), bad (orange, 21–40), mediocre (yellow, 41–70), good (green, 71–120), and very good (>120), noting that some years lack corresponding data.

This positive trajectory in the IBMWP index, transitioning from “Bad” to “Moderate” over the period, is attributed to a series of ecological interventions. This positive shift in the IBMWP index is plausibly attributed to a series of ecological interventions, encompassing sanitation measures that often improve water quality [47], and river restoration initiatives that augment habitat conditions, fortify existing species, and elevate environmental complexity and resilience [48,49].

Supporting studies corroborate the remarkable recovery of the Congost River and its encompassing basin over time [26], highlighting advances in various ecological indicators. Notable findings include an increase in the IPS (Specific Pollution Sensitivity) index (from 6 to 10), a rise in quality-associated avian species sightings from 5 during 1997–2022 to 15 between 2015 and 2017, and the identification of 9 out of Catalonia's 17 native amphibian species in the area. Reptile species count have also increased from 6 in 1997–2012 to 11 in 2013–2017. Additionally, the once-declared extinct crayfish species, *Austropotamobius pallipes*, re-emerged in 2008.

The Observatori RIVUS project further documents significant milestones [24,25], such as the re-colonization of the Eurasian Otter *Lutra lutra* in the Besòs river Basin in 2004 and its first recorded reproduction in the Congost River in 2020 since its earlier disappearance. This collective evidence underscores a significant ecological resurgence in the Congost River and its basin over the studied period.

### 3.2 Evolution of public perceptions of the Congost River's environment. Comparative study 2010–2022

Table 2 presents the outcomes of the Mann-Whitney *U* test comparing public perceptions related to the Congost River's environment across surveys from 2010 to 2022. The 2022 Survey revealed that users largely felt the river's water quality remained unchanged, with a median perception of 1 (IQR: 0.5) on a scale where 0.25 signifies “Worsened a lot”, 1 indicates “Stayed the same”, and 1.75 denotes “Improved a lot”. This represents a shift from the 2010 survey's median of 1.25 (IQR: 0.44), suggesting a perceived decline in water quality (CLES = 62.79, *p-value* = $7.89\times 10^{-5}$). A similar trend is noted in perceptions about riparian vegetation, with the median perception shifting from 1.25 (IQR: 0.5) in 2010 to 1 (IQR: 0.5) in 2022 (CLES = 58.00, *p-value* = 0.004).

**Table 2:** Comparative perceptions of the Congost River's water quality and riparian vegetation: 2010 vs. 2022. Results from Mann-Whitney U tests. Response levels of “Worsened a lot”, “Worsened considerably”, “Worsened a little “Stayed the same”, “Improved a bit”, “Improved considerably”, “Improved a lot” were replaced by numeric values of 0.25, 0.5, 0.75, 1, 1.25, 1.5 and 1.75, respectively.

	“How do you perceive the changes in water quality and riparian vegetation of the river over the past years?”					
	Year (n)	Mann-Whitney Group	Median (IQR)	U statistic	CLES	Significance of the difference
Water quality	2010 (n = 150)	Group 1	1.25 (0.44)	10550	62.79	*p-value* = $7.89\times 10^{-5}$
	2022 (n = 111)	Group 2	1 (0.5)			
Riparian vegetation	2010 (n = 150)	Group 1	1.25 (0.5)	9745	58.00	*p-value* = 0.0041
	2022 (n = 108)	Group 2	1 (0.5)			

As illustrated by Table 3, the Congost River's environment continues to be highly valued, with the 2022 survey recording a significant rise in median importance score to 7 out of 10 (IQR: 3), up from 6 (IQR: 2) in 2010 (CLES = 32.62, *p-value* = $1.63\times 10^{-6}$). However, perceptions related to Can Cabanyes remained statistically unchanged.

**Table 3:** Evaluation of the Congost River's environmental importance among Granollers' Tourist Attractions: 2010 vs. 2022. Results from Mann-Whitney U tests. Ratings were based on a scale of 1–10.

	“On a scale of 1–10, with 1 being not important at all and 10 being extremely important, how would you rate the importance of the Congost River's environment and Can Cabanyes among all the tourist attractions in Granollers?”					
	Year (n)	Mann-Whitney Group	Median (IQR)	U statistic	CLES	Significance of the difference
The Congost River's environment	2010 (n = 150)	Group 1	6 (2)	5138.5	32.62	*p-value* = $1.63\times 10^{-6}$
	2022 (n = 105)	Group 2	7 (3)			
Can Cabanyes	2010 (n = 78)	Group 1	6 (2.5)	2862.5	48.28	*p-value* = 0.7128
	2022 (n = 76)	Group 2	7 (2)			

The data also highlight a concerning trend: a growing number of respondents are unaware of recent restoration interventions. In 2022, 71.42 % of respondents were unaware of any recent restoration activities, marking a 17 % increase from 54.66 % in 2010 $\left(X^{2}=6.94,df=1,p-value=.0084\right)$. Similarly, unfamiliarity with species recovery efforts in the river ecosystem rose by 21 %, from 54.66 % in 2010 to 75.89 % in 2022 $\left(X^{2}=11.59,df=1,p-value=.0006\right)$.

Finally, “water pollution” continues to be the most perceived relevant environmental problem (22.01 % in 2022, only 3.16 points less than in 2010), other environmental concerns have evolved. Concerns about “riverside degradation” have increased from 9.98 % in 2010 to 14.37 % in 2022, and apprehensions about “low water flow” rose from 10.69 % to 18.35 %. Conversely, worries regarding “improper waste disposal” and “mosquito infestation” declined, from 23.28 % to 13.78 % in 2010 to 19.57 % and 9.48 % in 2022, respectively $\left(X^{2}=24.65,df=7,p-value=.0008\right)$.

### 3.3 Main differences regarding public perceptions among sociodemographic groups of users

This section delves into the significant differences in perceptions among various sociodemographic groups of users. We present the median (M.), interquartile range (IQR), common language effect size (CLES), as well as unadjusted and Bonferroni-adjusted p-values, which considers each independent variable as separate families of comparisons (see Table 4). In addition, we present mean ratings for each statement based on the collective responses from all 112 participants in the 2022 Survey, which represent non-segmented data. All these comprehensive results are shown in Fig. S2 of the Supplementary materials.

**Table 4:** Pairwise comparisons of nominal sociodemographic variable levels using Mann-Whitney *U* test. Scale from 1 (“strongly disagree”) to 5 (“strongly agree”). For each comparison, the structure is consistently presented as “Group 1 - Group 2”. “Group 1” serves as the reference point for the calculated common language effect size (CLES) in every pair. Thus, CLES values represent the probability that a randomly selected observation from “Group 1” will rank higher than one from “Group 2”. Statistical significance is indicated by a letter followed by a number: The letter denotes the significance level with values; a = *p-value* < 0.05; b = *p-value* < 0.005; and c = *p-value* < 0.0005. The number differentiates between unadjusted (1) and Bonferroni-adjusted p-values (2), which considers each independent variable as a separate family of comparisons.

Statement	Sampling point:		Employment status:			Access to river's environment:	Environmental volunteering participation:		
	Can Cabanyes - Parc Firal	Palou - Parc Firal	full-time working - not seeking e.	retired - seeking employment	retired - full-time working	on foot - vehicle	no - yes	no - yes, but not currently	yes, but not currently - yes
“Bad Odours”			4 (1) - 5 (0), 0.22^a₁^		4 (1) - 4 (1), 0.66^a₁^				
“River Management Lacking”				4 (1) - 3 (2), 0.72^a₁^	4 (1) - 4 (1), 0.63^a₁^				
“Dirtiness in the River”					4 (1) - 4 (1), 0.67^b₁a₂^		4 (1.75) - 2 (0.5), 0.87^a₁^		
“River-Walking Unsafety”							2 (1) - 4 (0), 0.13^a₁^		2 (0) - 4 (0), 0.00^a₁^
“Improved Flood Management”				4 (0) - 3 (1), 0.74^a₁^	4 (0) - 3 (1), 0.72^c₁b₂^				
“Limited River Knowledge”						4 (1) - 2.5 (1), 0.76^b₁a₂^			
“River Landscape Beauty”					4 (0) - 3.5 (1), 0.65^a₁^				
“River Quality Improvement”						3 (2) - 4 (1.5), 0.25^a₁a₂^			
“Recreational and Sports Opportunities”		4 (1) - 4 (0), 0.68^b₁b₂^							
“Cultural Heritage Connection”						4 (0) - 4.5 (1), 0.27^a₁a₂^			
“Nature and Children”		4 (1) - 4 (0), 0.66^b₁a₂^							
“Promoting Native Species”		4 (1) - 4 (0), 0.67^b₁a₂^							
“I Appreciate Biodiversity”	4 (1) - 4 (1), 0.73^a₁^	4 (1) - 4 (1), 0.67^b₁a₂^							
“WWTP and Water Quality”						4 (0) - 4.5 (1), 0.31^a₁^			
“Environmental Crisis Overstated”								2 (2) - 4 (1), 0.14^a₁^	

#### 3.3.1 Nominal sociodemographic variables

Table 4 presents post-hoc Mann-Whitney *U* test outcomes regarding nominal sociodemographic variables, following the rank-based Kruskal-Wallis tests. These tests were intended to discern statistically significant differences across sociodemographic groups based on their responses to the thirty statements.

Pairwise comparisons in the table are consistently formatted as Group 1 - Group 2. For instance, “Can Cabanyes” versus “Parc Firal” depicts “Can Cabanyes” as Group 1. This structure simplifies the interpretation of the common language effect size (CLES): CLES values denote the probability of an observation from Group 1 scoring higher than one from Group 2.

A critical observation from our analysis is how the sampling point significantly influences responses to environmental statements. Particularly, responses from Parc Firal, a heavily urbanized area, contrast with those from more naturally surrounded areas like Can Cabanyes and Palou. For instance, participants at Can Cabanyes, surrounded by richer natural features, showed stronger appreciation for biodiversity compared to those at Parc Firal - this was quantified by a median rating of 4 (IQR: 1) for both groups, but with a higher probability (CLES = 0.73, *p-value* = 0.018) of a more positive rating at Can Cabanyes. Similarly, respondents in Palou, another less urbanized zone, consistently showed greater support for environmental appreciation across statements such as “Recreational and Sports Opportunities”, “Nature and Children”, and “Promoting Native Species”, underscoring a trend where less urbanization correlates with higher environmental appreciation.

In examining the impact of employment status on environmental perceptions, distinct differences emerge, particularly between full-time workers and retirees. Full-time workers tend to be less sensitive to issues like “Bad Odours” compared to those not currently seeking employment, potentially due to their daily routines and reduced exposure to the environment (M. (IQR): 4 (1) - 5 (0), CLES = 0.22, *p-value* = 0.0292). Conversely, retirees exhibit more pronounced concerns about river management and flood control, frequently criticizing perceived inadequacies. Their responses, which often reflect a desire for enhanced management measures such as “River Management Lacking” and “Improved Flood Management”, suggest an acute awareness of and engagement with the river's current state compared to other groups. This heightened sensitivity and critical perspective may stem from their ability to compare the river's current condition with less favourable past scenarios, thereby providing them with a unique vantage point to incisively critique and advocate for continued improvements. Despite their criticisms, retirees also express a strong appreciation for the river's natural beauty, indicating a complex but ultimately positive relationship with the river environment.

The analysis of how different modes of accessing the river influence perceptions highlights clear distinctions between car users and pedestrians. Car users, who generally plan specific trips to visit river sites, showed a more comprehensive understanding of river-related issues. This was evident from their lower scores on “Limited River Knowledge” (M. (IQR): 4 (1) - 2.5 (1), CLES = 0.76, *p-value* = 0.0036), suggesting a higher level of river-related knowledge compared to pedestrians. Additionally, their perceptions of “River Quality Improvement” were more favourable (M. (IQR): 3 (2) - 4 (1.5), CLES = 0.25, *p-value* = 0.0073), possibly reflecting the focused nature of their visits which might be less frequent but more intentional than those of pedestrians.

Moreover, car travellers also rated “Cultural Heritage Connection” and “WWTP and Water Quality” higher than pedestrians, indicating that the purpose and nature of their visits might allow them to perceive the river's cultural and ecological aspects more positively. These differences suggest that expectations and experiences, shaped by the mode of access to the river, play a significant role in influencing perceptions.

Participation in environmental volunteering also influenced perceptions of river conditions. Individuals not engaged in volunteering perceived “Dirtiness in the River” more critically than volunteers (M. (IQR): 4 (1.75) – 2 (0.5), CLES = 0.87, *p-value* = 0.0236). This may suggest non-volunteers are possibly less aware of ongoing restoration efforts or maintain different expectations of cleanliness. Regarding safety, non-volunteers reported feeling safer, reflected in their less frequent agreement with the statement “River-Walking Unsafety” (M. (IQR): 2 (1) – 4 (0), CLES = 0.13 *p-value* = 0.0185). Interestingly, past volunteers also reported feeling safer than current volunteers (M. (IQR): 2 (0) – 4 (0), CLES = 0.00 *p-value* = 0.0468), suggesting that active involvement may heighten awareness of potential risks not apparent to the broader community or those less frequently involved.

Contrary to expectations, our findings reveal that past volunteers were more likely than non-volunteers to view the narrative surrounding the current environmental crisis as overstated (M. (IQR): 2 (2) - 4 (1), CLES = 0.14, *p-value* = 0.0299). This surprising trend suggests a complex relationship between past volunteer experiences and their current perceptions of environmental urgency. While this does not negate the reality of environmental challenges, it may reflect a critical perspective on environmental discourse, potentially shaped by personal experiences or perceptions of progress in environmental initiatives. Such results underscore the need for ongoing dialogue and education to align perceptions with scientific consensus on environmental issues.

Intriguingly, gender did not show a significant effect on any of the surveyed statements, indicating that perceptions regarding river conditions and management might transcend gender differences. Out of 21 statistically significant outcomes initially identified, 9 retained their significance after applying a Bonferroni correction, which adjusts for multiple comparisons to reduce the likelihood of false positives. This statistical approach reaffirms the robustness of the key findings. For a detailed breakdown of these results, refer to Table 4 for a comprehensive overview.

#### 3.3.2 Quantitative and dichotomous sociodemographic variables

Table 5 outlines the significant effects of quantitative sociodemographic variables on statement mean ratings, as determined by linear models. Each regression coefficient indicates the expected change in the statement's mean rating for a one-unit increase in the variable. We conducted the regression models using each sociodemographic variable independently, without controlling for other sociodemographic factors.

**Table 5:** Statistically significant coefficients $\left(\beta_{i}\right)$ obtained from the regression models with significant effects on the statements. Statistical significance: a = *p-value* < 0.05; b = *p-value* < 0.005; c = *p-value* < 0.0005.

Statements	Quantitative sociodemographic variable					
	Age [<77]	Education level	Household income	Residence surroundings	Num. of weekly visits	Num. of years familiar with.
“Bad Odours”				0.298^a^		
“Dirtiness in the River”		−0.207^b^		0.394^b^	0.093^a^	
“Inaccessible Riverside Paths”	−0.014^a^					
“River-Walking Unsafety”						−0.016^a^
“Improved Flood Management”		−0.115^a^		0.275^a^	0.086^a^	
“Disbelief in River Clean-up”	0.011^a^					
“Limited River Knowledge”		−0.246^c^	−0.445^b^			
“Inaccessibility of the River”	−0.014^a^					
“River Quality Improvement”		0.167^a^			−0.093^a^	
“Unnoticed River Interventions”	−0.015^b^					
“Nature and Children”					−0.099^c^	
“River Risks”		−0.224^b^				
“I Appreciate Biodiversity”	0.017^c^				−0.063^a^	
“WWTP and Water Quality”	0.009^a^					
“Environmental Crisis Overstated”	0.015^a^	−0.253^b^				

Among the analysed factors, “Age [<77]”, “Education level”, and “Num. of weekly visits” were found to have the most significant impact. For the “Age” factor, we considered observations below 77 years, excluding three influential data points over 77 years. The results reveal a positive correlation of “Age [<77]” with statements reflecting environmental engagement and perceptions. This included a strong disbelief in the river needed to be cleaned (β = 0.011, *p-value* = 0.0344), greater appreciation for biodiversity (β = 0.017, *p-value* = $6.31\times 10^{-5}$), and more favourable perceptions of water treatment and quality (β = 0.009, *p-value* = 0.0234). Age also correlated positively with the notion that the environmental crisis is overstated (β = 0.015, *p-value* = 0.0398). Conversely, negatively correlated with perceptions of river inaccessibility, suggesting that older individuals are less likely to perceive riverside paths as inaccessible (β = −0.014, *p-value* = 0.0112), and less likely to report a lack of river access (β = −0.014, *p-value* = 0.0288). Interestingly, older participants also indicated a belief that the general public is more aware of interventions made to improve the river, contrary to the statement “the general public is unaware of interventions made to improve the river” (β = −0.015, *p-value* = 0.0041). This suggests that older individuals, possibly having witnessed numerous changes and improvements firsthand, perceive a greater public awareness of environmental efforts, reflecting an informed perspective on river health progress.

The analysis of “Education level”, treated as ordinal and excluding responses classified under “Others” and “No education”, revealed distinct correlations with perceptions of river health and management. Higher education positively influenced the perception of “River Quality Improvement” (β = 0.167, *p-value* = 0.0172), suggesting that more educated individuals recognize and appreciate efforts to enhance river quality.

Conversely, a higher education level was associated with more critical views on several aspects of river management and conditions. Educated individuals were less likely to perceive the river as dirty or worsening over time (β = - 0.207, *p-value* = $9.23\times 10^{-4}$), and they expressed scepticism towards the adequacy of flood management (β = −0.115, *p-value* = 0.0417). Additionally, this group reported more accurate knowledge of natural river processes, contrary to the notion of limited knowledge, as indicated by a negative correlation with the statement about limited river knowledge (β = −0.246, *p-value* = $2.66\times 10^{-5}$). Importantly, educated individuals were less likely to consider “River Risks” (β = −0.224, *p-value* = 0.0021), and the environmental threats exaggerated (β = −0.253, *p-value* = 0.0012), further indicating that higher education levels may foster a more realistic and informed view of environmental processes and risks.

The variable “Number of weekly visits” also demonstrated significant associations with perceptions of river conditions. Interestingly, more frequent visits correlated positively with “Improved Flood Management” (β = 0.086, *p-value* = 0.0058), suggesting that individuals who visit the river more often may be more attuned to specific issues and improvements in flood management.

On the other hand, more frequent visits were associated with more critical views on several aspects. There was a positive correlation with perceptions of “Dirtiness in the River” (β = 0.093, *p-value* = 0.0109) and negative correlation with “River Quality Improvement” (β = −0.093, *p-value* = 0.0193), indicating that regular visitors might have higher standards or expectations for water quality that are not being met. Additionally, frequent visitors reported less agreement for “Nature and Children” (β = −0.099, *p-value* = 0.0001) and less appreciation for biodiversity (β = −0.063, p-value = 0.0304). This critical stance may reflect a strategic perspective where visitors hold conservation efforts to higher standards to ensure that improvements continue, thereby preserving and enhancing the natural spaces they frequently enjoy.

Our analysis also revealed that the ordinal variable “Residence surroundings”, indicating the respondent's perception of the natural environment around their homes, positively correlated with statements such as “Bad Odours” (β = 0.298, *p-value* = 0.0095), “Dirtiness in the River” (β = 0.394, *p-value* = 0.0024) and “Improved Flood Management” (β = 0.275, *p-value* = 0.0138), suggesting that those living in more natural environments might be more sensitive to these issues. In contrast, “Household income” was negatively correlated only with “Limited River Knowledge” (β = −0.445, *p-value* = 0.0014), indicating that higher incomes levels might be associated with less perceived knowledge about river processes.

Additionally, the variable “Num. of years familiar with”, denoting the respondent's familiarity duration with the Congost River's environment, negatively influenced perceptions of safety, as seen in the correlation with “River-Walking Unsafety” (β = −0.016, *p-value* = 0.0307). This relationship suggests that individuals with many years of familiarity with the river may express concerns about river-walking safety not solely because they feel unsafe, but rather as a strategic way to draw attention to areas that need improvement. This approach mirrors that of more frequent visitors who, as identified in our findings, may adopt a critical stance on various aspects of river conservation to ensure continued restoration efforts.

Table 6 outlines the outcomes from the Mann-Whitney *U* test that compared responses between individuals who live with children under 18 years old and those who do not (Group 1 = respondents not living with children under 18 years, and Group 2 = respondents living with them). The results indicate that respondents without children under 18 years generally reported higher median values for most statements, suggesting that their perceptions might differ significantly in regards to various aspects of river environment and management.

**Table 6:** Differences in perceptions based on living with children under 18 years: results from the Mann-Whitney *U* Test. Scale from 1 (“strongly disagree”) to 5 (“strongly agree”). For CLES interpretation, people living without children under 18 years is considered Group 1, while those living with them is considered Group 2. Sample sizes: *N* “Children <18years: no” = 90, *N* “Children <18 years: yes” = 22.

	Levels of the variable: Group 1 = no; Group 2 = yes	Mean (IQR)	CLES	Significance of the difference
“Inaccessible Riverside Paths”	no	2 (1)	0.34	*p-value* = 0.0161
	yes	3 (1.75)		
“Improved Flood Management”	no	4 (1)	0.65	*p- value* = 0.0175
	yes	3 (1)		
“Recreational and Sports Opportunities”	no	4 (1)	0.71	*p- value* = 0.0007
	yes	4 (0.75)		
“Cultural Heritage Connection”	no	4 (0)	0.62	*p- value* = 0.0439
	yes	4 (0)		
“Nature and Children”	no	4 (1)	0.70	*p- value* = 0.0004
	yes	4 (0)		
“Promoting Native Species”	no	4 (1)	0.67	*p- value* = 0.0050
	yes	4 (0)		
“Shared Responsibility for Water Conservation”	no	4.5 (1)	0.63	*p- value* = 0.0327
	yes	4 (0.75)		
“I Appreciate Biodiversity”	no	4 (0.75)	0.68	*p- value* = 0.0045
	yes	4 (1)		
“WWTP and Water Quality”	no	4 (1)	0.65	*p- value* = 0.0134
	yes	4 (1)		

However, an exception was noted in the perception of “Inaccessible Riverside Paths”. Here, respondents not living with children scored a median of 2 (IQR: 1), indicating less concern about accessibility, whereas those with children under 18 reported greater concerns, with a median score of 3 (IQR: 1.75) (CLES = 0.34, *p-value* = 0.0161). This suggests that living with children may heighten sensitivity to issues of accessibility in river environments, likely due to the increased importance of safe and accessible recreational spaces for families.

## 4 Discussion

The primary findings of this study indicate a marked appreciation for the Congost River as a valuable site in Granollers, resonating with the global narrative emphasizing the importance of natural spaces, especially in light of the COVID-19 pandemic [50,51]. Such green spaces function as refuges from urban pressures and are essential for mental health during challenging periods [52,53]. The increased attention to these areas, influenced by events from 2010 to 2022, reflects a global shift towards prioritizing green spaces.

Interestingly, this rising appreciation did not translate into an enhanced perception of water quality and riparian vegetation among respondents. A majority indicated that these attributes remained unchanged over the observed period. This perception aligns with the persistently low Riparian Quality (QBR) index values [26]. The QBR index, which evaluates both vegetation and riverbank hydromorphological quality as delineated by Munné (et al., 2003) [54] showed no significant improvement despite growing public advocacy for vegetation augmentation. Typically, urban rivers characterized by infrastructural developments on their margins exhibit diminished QBR values. This underscores the complex urban dilemma of improving riparian habitats, a reflection of a broader challenge in urban ecological restoration where infrastructure often impedes ecological progression [55].

While the IBMWP index indicates enhancements in river quality, prevailing public sentiment contends that there has been no discernible change. This perceptual inertia, highlighted by Okumah et al. [56] and Steinwender et al. [10] suggests that direct sensory experiences, such as colour, odour, and visual aesthetics, potentially eclipse nuanced biological markers in informing public perception. On a more extensive scale, it is evident that the general population tend to prioritize immediate sensory inputs over complex ecological metrics. This underscores the inherent challenge of harmonizing objective scientific assessments with the subjectivity of human experiences [57].

Past narratives continue to dominate the discourse around “water pollution”, even amidst evident improvements in contaminant data [26]. This lingering influence of past environmental conditions on present-day perceptions is a global phenomenon, suggesting that long-standing perceptions can potentially impede proactive community engagement [58].

Our findings diverge from previous works by Jeon et al. [59] and Steinwender et al.[10]. Contrary to their findings of a positive correlation between public perceptions and measured water quality, our study unveils a significant discrepancy. This disparity underscores the overarching challenge in environmental science - translating objective scientific data into comprehensible narratives for the broader community. Such perception-data mismatches can inadvertently bias local and global environmental decisions, emphasizing the urgent need for effective community education and engagement [60].

One central explanation for the observed discrepancy between public perceptions and the river's ecological quality lies in the weight the general public places on aesthetic features [61]. This aesthetic lens can, at times, blur the lines between what is visually appealing and what is ecologically sound. Junker and Buchecker [62] provide insight into this, highlighting the potential disconnection between aesthetic appreciation and actual ecological values. Gobster et al. [63] further delve into this by pointing out an inherent human tendency: equating visually pleasing environments with ecological health. However, this relationship is not straightforward. Solely gauging an ecosystem's health on its appearance can lead to skewed perceptions [64]. To foster a more accurate understanding of river health, there is a pressing need for improved environmental literacy and a deeper appreciation of riverine functions [65]. In our study, we have found that older people, more educated, and with current involvement in environmental volunteering were more likely to be aligned with scientific ecological data.

Emphasizing cultural sustainability, as advocated by Nassauer [66], becomes critical in this scenario. The idea is to shape public attitudes, values, and knowledge, especially since individuals often resonate more with the recognizable. Our study, aligned with findings from Ioana-Toroimac et al. (2020), which uncover a pronounced knowledge gap among users, with many revealing limited insights into river processes. Such findings underscore the critical importance of proactive environmental education and community engagement, reflecting a global consensus [67].

The Congost River, in the eyes of its users, emerges as a symbolic representation of Granollers city. It is not just a natural landmark but also a hub for economic, recreational, and cultural activities. As Ryan et al. [68] points out, such multifaceted associations reiterate the potential of leveraging these positive perceptions for community-led conservation initiatives.

Our study sheds light on the intricate relationship between an individual's surroundings, their frequency of interaction with the environment, and their subsequent perceptions. Specifically, those surrounded by more natural landscapes appear more attuned to aspects like “bad odours”, while regular river visitors seem more critical of its state. Prior research indicates that a profound connection with nature fosters pro-environmental behaviour [69]. Consequently, due to their regular interactions with the environment, both individuals living in natural settings and regular river visitors are likely to adopt a more critical and discerning viewpoint, championing continuous environmental investment. Intriguingly, this critical viewpoint stemming from heightened sensitivity among frequent visitors might directly contribute to their unexpected underappreciation of biodiversity, even though biodiversity is recognized as a primary attraction in natural spaces [70]. In contrast, those involved in environmental volunteering or possessing advanced education typically have more positive perceptions of the river's state. These observations align with global studies suggesting that educational background significantly influences environmental perceptions [71].

Age emerges as a crucial determinant in perceptions, especially concerning accessibility and biodiversity appreciation. Our study found that older individuals appreciate biodiversity more, possibly because they remember the river's conditions being worse in the past. This recollection resonates with other studies that underline the historical and aesthetic values older populations associate with natural habitats [72,73].

In a nutshell, this study illuminates the complex web of factors shaping public perceptions of the Congost River. The findings underscore the imperative to bridge the gap between objective scientific data and subjective human experiences, reiterating the critical role of community engagement, education, and strategic communication in environmental conservation.

### 5.1 Limitations

Future research on public perceptions of ecologically restored environments should focus on identifying the most valued elements for respondents. Our study found that river environment users generally do not perceive that the ecological quality has improved over time and that this perception varies across sociodemographic groups. However, we were unable to uncover the specific environmental elements that users deem crucial when assessing a natural environment's ecological quality. By incorporating questions that elicit this information in future studies, it could be better understood whether there is a disconnect between the perceptions of managers and general users or if each group is utilizing different metrics to evaluate the restoration process.

While our research provides valuable insights, it underscores a global challenge in environmental studies: understanding which ecological facets resonate most with the public. Future endeavours, by delving into this, can not only refine local restoration processes but also influence global strategies in environmental restoration.

## 5 Conclusions

Our comprehensive exploration of the Congost River and Can Cabanyes in Granollers acts as a detailed case study, illustrating the complex relationship between human perceptions and objective environmental metrics. Despite discernible improvements in the river's ecological health, as evidenced by various biological indicators, a perceptual discord remains among the river's diverse users. From this detailed analysis, several pivotal conclusions emerge.

The Congost River, over the past three decades, has experienced notable advancements in its biological and physicochemical health. However, despite this progress, riparian vegetation, as gauged by the QBR index, continues to be a focal concern.

The esteem and recognition for the Congost river has seen a marked surge since 2010, echoing a global sentiment that accentuates the intrinsic value of natural environments.

A significant segment of the river's user population remains unconvinced of its ecological recovery, emphasizing the pressing need for enhanced communication strategies that bridge the gap between empirical evidence and public perception.

Older individuals, those with advanced education levels and environmental volunteers often align their insights more closely with empirical data, underscoring their nuanced understanding of the river's evolving narrative.

Residents with a richer natural surroundings and frequent river visitors manifest heightened sensitivity to the river's aesthetic and potential bad odours.

In conclusion, our study not only dissects the interplay between community perceptions and objective environmental indicators but also aligns these findings with overarching global trends. As conservation efforts globally gain traction, our findings emphasize the paramount importance of community-driven initiatives, proactive education, and transparent communication in shaping cohesive ecological restoration visions.

## Data availability

The authors do not have deposited the data associated with this study into the publicly available repository. The authors do not have permission to share the data.

## Novelty and relevance statement

Our study presents original insights into the alignment (or lack thereof) between public perception and the ecological progress of river restoration. We have innovatively linked sociodemographic factors with environmental awareness and perception, revealing how various groups interpret ecological improvements. Our findings are crucial for environmental management, highlighting the need for effective communication strategies to bridge the perceptual gap in ecological restoration. This research is not only relevant to the Congost River but also offers broader implications for global environmental management practices, emphasizing the importance of incorporating community perspectives in environmental conservation and policy-making.

## CRediT authorship contribution statement

**Quim Zaldo-Aubanell:** Writing – review & editing, Writing – original draft, Visualization, Methodology, Investigation, Formal analysis, Data curation, Conceptualization. **Antoni Mas-Ponce:** Writing – review & editing, Methodology. **Guiu Asbert:** Visualization, Investigation. **Berta Clota:** Investigation. **Manel Isnard:** Methodology. **Lorenzo Proia:** Writing – review & editing, Validation. **Albert Bach:** Visualization. **Sònia Sànchez Mateo:** Writing – review & editing, Validation, Supervision, Resources, Project administration, Methodology, Funding acquisition.

## Declaration of generative AI and AI-assisted technologies in the writing process

During the preparation of this work the authors used Grammarly in order to proofread the manuscript. After using this tool/service, the authors reviewed and edited the content as needed and take full responsibility for the content of the publication.

## Declaration of competing interest

The authors declare the following financial interests/personal relationships which may be considered as potential competing interests: Quim Zaldo-Aubanell reports financial support was provided by Granollers City Council (10.13039/501100007601Horizon 2020, European Union, Project No. 887396). Quim Zaldo-Aubanell reports financial support was provided by Fundació RIVUS, in collaboration with Consorci Besòs Tordera.
