# Supplementary material for: Public perception vs ecological quality status: Examining the ecological restoration of the Congost River's Environment
Source: Heliyon. 2024 Jul 14;10(14):e34615. doi: 10.1016/j.heliyon.2024.e34615 (PMC11315083; doi:10.1016/j.heliyon.2024.e34615)
Supplement: Multimedia component 1 [file mmc1.docx]

**Supplementary Material**

**Figure S1a.** 2022 Survey 1^st^ Part. Sociodemographic questions used to gather the socioeconomic profile of respondents.

| **Age:** |
| --- |
|  |

| High school |  |
| --- | --- |
| Technical/vocational |  |
| College degree |  |
| Graduate degree |  |
| Master’s or higher |  |
| No education |  |
| Other |  |

**Education level**

| Yes |  |
| --- | --- |
| No |  |

**Do you live with any children younger than 18 years old?**

| Female |  |
| --- | --- |
| Male |  |
| Non-binary |  |
| Prefer not to say |  |

**What is your gender identity?**

| Less than 30% natural environment (trees, vegetation...) |  |
| --- | --- |
| Between 30% and 50% natural environment |  |
| More than 50% natural environment |  |

**How would you describe your residential area?**

**What is your current employment status?**

| Not seeking employment |  |
| --- | --- |
| Seeking employment |  |
| Retired |  |
| Part-time working |  |
| Full-time working |  |

**What is the approximate range of your household’s total annual income?**

| 0 – 21.000 € |  |
| --- | --- |
| 21.001 - 40.000 € |  |
| > 40.000 € |  |
| Prefer not to say |  |

**What is the municipality you currently reside in?**

**How do you primarily access the river’s environment?**

| On foot |  |
| --- | --- |
| Bike, scooter or similar |  |
| Motorbike |  |
| Car or other motor vehicle |  |

**How frequently do you visit the river environment per week?**

| Please indicate number |  |
| --- | --- |
| It’s the first time |  |

**How long have you been familiar with the river’s environment?** (Please indicate number of years)

**Have you ever participated in any environmental volunteer work?**

| Yes |  |
| --- | --- |
| No |  |
| Yes, but not currently |  |

**Figure S1b.** 2022 Survey 2^nd^ Part. Selected questions from the 2010 Survey.

|  | Water quality | Riparian vegetation |
| --- | --- | --- |
| Improved a lot |  |  |
| Improved considerably |  |  |
| Improved a bit |  |  |
| Stayed the same |  |  |
| Worsened a little |  |  |
| Worsened considerably |  |  |
| Worsened a lot |  |  |

**How do you perceive the changes in water quality and riparian vegetation of the river over the past years?**

**Are you aware of any restoration interventions that have been carried out in the past years?**

| Yes |  |
| --- | --- |
| No |  |

**What do you think should be done to improve the river’s environment?** (Choose up to 2 options or add your own)

| Remove exotic invasive species |  |
| --- | --- |
| Increase the native riparian vegetation |  |
| Control waste disposal and debris |  |
| Improve riverside walkways |  |
| Restrict development of infrastructure on the riverbank |  |
| Develop an education program on the Congost River |  |
| Improve water quality |  |
| ... |  |

| The Congost river’s environment | Can Cabanyes |
| --- | --- |
|  |  |

**On a scale of 1-10, with 1 being not important at all and 10 being extremely important, how would you rate the importance of the Congost River’s environment and Can Cabanyes among all the tourist attractions in Granollers?**

**Are you aware of any species that have recovered in the river ecosystem in recent years? If so, please specify.**

**What are your preferred options for the future state of the river environment?** (Select 1 or 2 options)

| River channel without riparian vegetation |  |
| --- | --- |
| Grass on both riverbanks |  |
| Increased herbaceous and bushy vegetation |  |
| Riparian forest |  |

**Are you aware of any invasive species present in the Congost river? If so, please specify.**

| **Flooding** |  |
| --- | --- |
| Water pollution |  |
| Riverside degradation |  |
| Presence of invasive species |  |
| Low water flow |  |
| Improper waste disposal |  |
| Mosquito infestation |  |
| Construction in the riverbed |  |

**Which of the following environmental issues do you think are currently affecting the Congost river? (Select up to 3 options)**

| **1 2 3 4 5** | **Strongly agree** | **Agree** | **Neutral** | **Disagree** | **Strongly disagree** |
| --- | --- | --- | --- | --- | --- |
| It’s easy to detect bad odours in the river’s environment |  |  |  |  |  |
| I perceive a lack of management in the current state of the river |  |  |  |  |  |
| I perceive the river to be dirty and worsening over time |  |  |  |  |  |
| The river is a symbol of the Granollers’s city |  |  |  |  |  |
| Riverside paths are not easily accessible due to overgrown vegetation |  |  |  |  |  |

**6
7
8
9
10**

**1
2
3
4
5**

**6
7
8
9
10**

**11
12
13
14
15**

**16
17
18
19
20**

**21
22
23
24
25**

**26
27
28
29
30**

**Figure S1c.** 2022 Survey 3^rd^ Part. Set of thirty statements gathering respondent’s attitudes towards the Congost River’s environment.

| I feel unsafe when walking near the river |  |  |  |  |  |
| --- | --- | --- | --- | --- | --- |
| I feel that the river’s surrounding is often overcrowded |  |  |  |  |  |
| Flooding should be better managed to minimize losses to crops and private property |  |  |  |  |  |
| I don’t often think about the river |  |  |  |  |  |
| I don’t believe the river needs to be cleaned |  |  |  |  |  |

**11
12
13
14
15**

| I have limited knowledge of natural river processes |  |  |  |  |  |
| --- | --- | --- | --- | --- | --- |
| I find the river landscape to be beautiful |  |  |  |  |  |
| I feel the river is not easily accessible |  |  |  |  |  |
| I perceive the river’s quality to have improved in recent years |  |  |  |  |  |
| The river serves as a vital ecological corridor for the habitat and wildlife of birds and other animals |  |  |  |  |  |

**16
17
18
19
20**

| The general public is unaware of interventions made to improve the river |  |  |  |  |  |
| --- | --- | --- | --- | --- | --- |
| The river plays a crucial role in supporting agriculture, industry, and economic activity |  |  |  |  |  |
| The river environment provides opportunities for sports and recreational activities |  |  |  |  |  |
| It is important for individuals to actively participate in shaping the future of the river |  |  |  |  |  |
| I enjoy living in close proximity to the river |  |  |  |  |  |

**21
22
23
24
25**

| The river connects us to our cultural heritage |  |  |  |  |  |
| --- | --- | --- | --- | --- | --- |
| The river environment offers opportunities for children to experience and appreciate nature |  |  |  |  |  |
| Investing in the restoration of the river is beneficial for our future |  |  |  |  |  |
| The river can pose health and environmental risks |  |  |  |  |  |
| Promoting native species is beneficial |  |  |  |  |  |

**26
27
28
29
30**

| Removing exotic vegetation from the river ecosystem is necessary, even if it has aesthetic value |  |  |  |  |  |
| --- | --- | --- | --- | --- | --- |
| Conserving water is a shared responsibility |  |  |  |  |  |
| I appreciate the diversity of plant and animal life in the river environment |  |  |  |  |  |
| Wastewater treatment plants play a crucial role in improving the water quality |  |  |  |  |  |
| The perception of an environmental crisis is overstated |  |  |  |  |  |


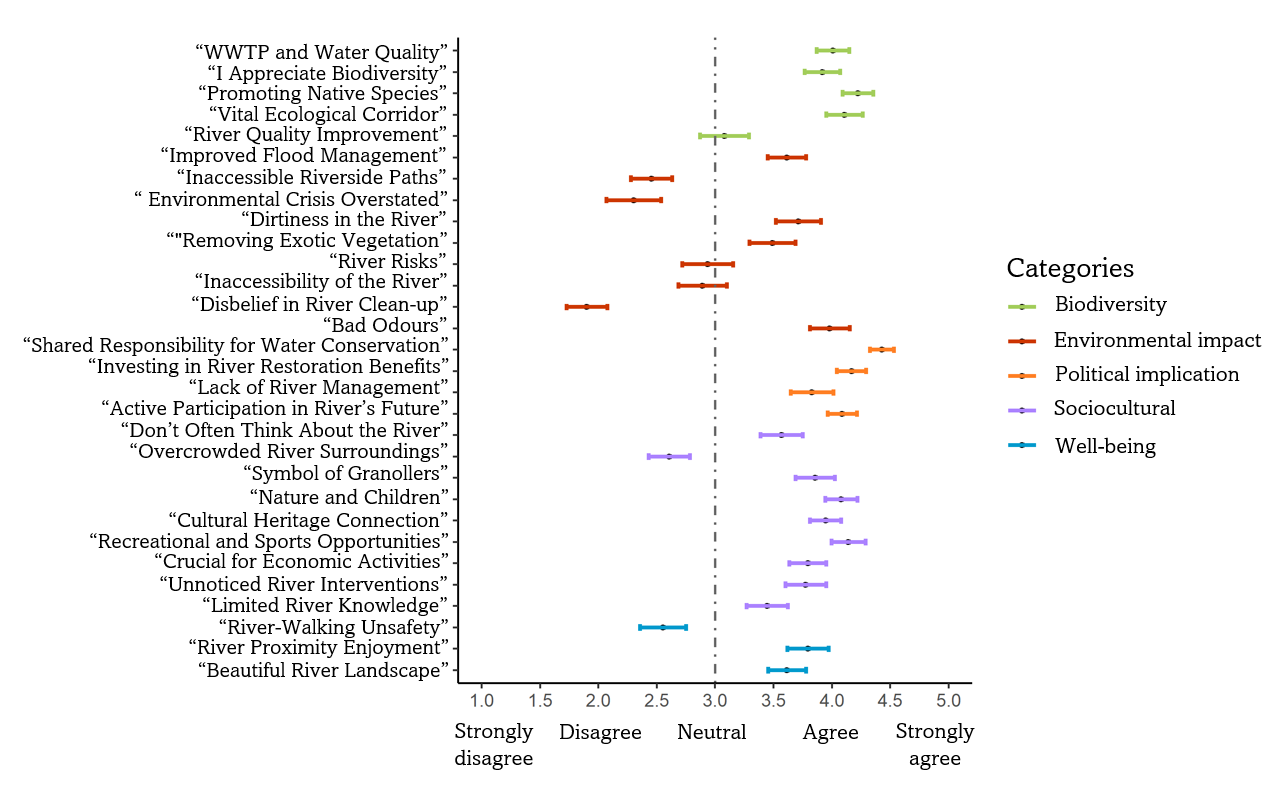
**Figure S2.** Mean ratings obtained from all responses without segmenting by socioeconomic profile (2022 Survey n=112). Scale from 1 (“strongly disagree”) to 5 (“strongly agree”). Statements are categorised into five groups: sociocultural, well-being, biodiversity, environmental impact and political implication
